# The effectiveness of preventive home visits on resilience and health-related outcomes among community dwelling older adults: A systematic review

**DOI:** 10.1371/journal.pone.0306188

**Published:** 2024-07-01

**Authors:** Dayang Balkis Ramli, Suzana Shahar, Sumaiyah Mat, Norhayati Ibrahim, Noorlaili Mohd Tohit

**Affiliations:** 1 Centre for Healthy Aging and Wellness, Faculty of Health Sciences, Universiti Kebangsaan Malaysia, Kuala Lumpur, Malaysia; 2 Public Service Department, Prime Minister’s Office, Putrajaya, Malaysia; 3 Department of Family Medicine, Faculty of Medicine, Universiti Kebangsaan, Bangi, Malaysia; Iran University of Medical Sciences, ISLAMIC REPUBLIC OF IRAN

## Abstract

**Background:**

This research aimed to assess the effectiveness of preventive home visits (PHVs) in enhancing resilience and health-related outcomes among older adults living in the community.

**Methods:**

A comprehensive literature search was conducted in nine databases (PubMed, MEDLINE, CINAHL, Embase, Emcare, Web of Science (WOS), Scopus, PsycINFO and Cochrane Library. The search was undertaken between March 15 and 31, 2022 with subsequent updates performed on October 15, 2023 and April 10, 2024. This review also included grey literature sourced via Google, Google Scholar and backward citation searches.

**Results:**

Out of 5,621 records, 20 articles were found to meet the inclusion criteria with a total of 8,035 participants involved and the mean age ranged from 74.0 to 84.4 years. Using McMaster Critical Review Form for Quantitative Studies, we ascertained that the studies included in our analysis had moderate to high levels of quality. In addition to health-related outcomes, PHV interventions were also conducted to evaluate psychological effects (16 studies) and social outcomes (seven studies). Five studies conducted financial assessment to evaluate the costs of health and social care utilisation during PHV interventions. Regarding the results of the review, seven studies showed favourable outcomes, five indicated no effect and eight had equivocal findings. Only one study assessed resilience and determined that PHV had no effect on the resilience of the subjects.

**Conclusion:**

This review found that the effectiveness of PHV interventions was uncertain and inconclusive. PHV interventions often prioritise health-related objectives. The incorporation of a holistic approach involving psychosocial health into PHV interventions is relatively uncommon. Due to the paucity of research on resilience as PHV outcome, we are unable to draw a conclusion on the effectiveness of PHV on resilience. Resilience should be prioritised as a psychological assessment in the future development of comprehensive PHV interventions, as it enables older adults to adapt, manage, and respond positively to adversities that may arise with age. Performing financial analysis such as costs and benefits analysis to incorporate the return on investment of PHV interventions is an added value for future research on this topic.

**Clinical trial registration:**

PROSPERO registration number: CRD42022296919.

## Introduction

The global older population is expanding rapidly as a result of technological advancements in healthcare and social services, leading to increased life expectancy. Concurrently, we are confronted with the issue of low fertility rates worldwide. The population of older adults is projected to grow by 4% from 1 billion in 2020 to 1.4 billion in 2030. In addition, it is expected that the percentage of those aged 80 years and older will triple throughout the same time frame [1]. The demographic transition has had a significant impact on our daily lives, resulting in both promising opportunities or challenging obstacles. The increasing number of older adults may affect productivity and economic growth due to the shrinking workforce and the increase of semi-retired and retired citizens [2, 3].

The demand for public health and social care services also increases as older adults experience age-related diseases and disabilities [4, 5]. Besides non-communicable diseases and old age-related diseases such as dementia and sarcopenia [6], 46% of the older adults experienced disability during their old age [7, 8]. Increases in old-age-related disability and illness have an impact on public healthcare provision and expenditure, human resource mobilisation and intergenerational challenges such as caregiver burden among family members [8–11].

It is essential to focus on maintaining good health in older adults in order to improve their overall well-being and quality of life (QoL). Advocating healthy ageing not only has a positive impact on the older adult population but also enhances the family institutions and communities by fostering the sharing of intergenerational care, knowledge and resources. Providing a safe and secure environment for older adults to feel protected, happy, independent, mobile and comfortably ‘age in place’ is a shared responsibility that involves interventions with a holistic approach and requires multisectoral commitment for its implementation [12].

In response to the adverse health risks associated with an ageing population, health promotion and disease prevention either domain-specific or multidimensional complex interventions have become priorities in promoting healthy ageing [11, 13]. Reliance on institutionalised healthcare services alone is insufficient due to high demand and limited resources. Therefore, preventive approaches such as preventive home care services become the alternative to close the gap of the dearth of resources in providing health care services for the rising numbers of older adults on top of the general population. The structured preventive home care service is commonly known as preventive home visit (PHV) or alternatively described as home-based nursing care, an in-home visit or simply a home visit.

PHV has been introduced decades ago and widely implemented in the West, particularly in the Nordic countries [14], Canada, Australia and Japan [15–18]. Based on its implementation and challenges, the United Kingdom discontinued the PHV programme [18] while the United State did not recommend PHV be implemented for the whole older adult population in the country [15]. This is due to the uncertain and inconclusive evidence of PHV effectiveness in improving older adults’ health [18–20]. The debate on the effectiveness of PHV is still ongoing and the rise of PHV research shows that more evidence of the effectiveness of PHV is essential to ensuring that the efforts spent on the programme are worth the benefit to the targeted beneficiaries.

PHV intervention includes the health assessment, conducted periodically at participants’ homes by focusing on specific needs of the older adults. The visit is conducted either by individuals (commonly by public health or district nurses) or a group of multidisciplinary professionals in the geriatric and gerontology fields such as nurses, physiotherapists, occupational therapists, social workers, dietitians, pharmacists or any other relevant professionals [14, 15, 17, 18, 21]. PHV also involves home environment assessments for fall risks and safety evaluations.

PHV as a disease prevention aids in slowing functional and cognitive decline and enables older adults to maintain or improve their QoL [14, 22], enhances autonomy, particularly in younger age groups and promotes independent living and self-care [18, 21, 23].

Several PHV interventions involved psychological assessments and social participation. Social participation among older adults with family members and the community around them enhanced their QoL by increasing their sense of control (SoC), life satisfaction and coping mechanisms [24–27] and by reducing loneliness and stress [28–30]. In recent years, particularly during the COVID-19 pandemic, the significance of social engagement and social connection has become more apparent [28–31].The decline in either psychological or social status or both, affected health condition of the older adults [31–34].

The psychosocial health of an individual is highly influenced or represented by their level of resilience. In a volatile and fast-paced society, resilience has been one of the most important health indicators for older adults [10]. Resilience varies based on protective factors and positive adaptation, as well as the severity of the context of adversity faced by the individual. These factors are interrelated and escalate from the individual level to the family, the societal context and finally to the higher context, the governance of a country [35–37].

Resilience is the capacity to adapt in the face of adversity, trauma, tragedy, hardship or threats [38–41] by utilising protective factors as positive adaptation in harnessing the adversity [24, 37, 42–45]. Following scholarly work and debates on the definition of resilience, the American Psychological Association has redefined the previous definition of resilience as “the process and outcome of a successful adaptation to difficult or challenging life experiences, Resilience is a complex phenomenon whose causes include biological, psychological, social and cultural contexts [37] that emerge throughout a person’s lifetime as either a characteristic, a process, or an outcome [46]. A resilient individual owns a mental, emotional and behavioural flexibility and is able to adjust to response to the external and internal demands” [47]. In general, older adults are more resilient than younger adults. Sociodemographic and health-related characteristics may have a greater impact on individuals’ resilience than age. Despite growing research on age-specific resilience interventions, the definition, assessment methods, multi-stakeholder’s involvement and the effectiveness remain contentious [10, 24, 35, 43].

Resilience can be promoted and strengthened through PHV intervention by integrating health, psychology and social components with the involvement of individuals and multisectoral collaboration that can spur dynamic interactions among the beneficiaries and care providers. This holistic approach is essential to be further explored among community health and preventive medicine researchers [48–50]. While a plethora of studies have examined the effects of PHV interventions on health and psychological outcomes, little is known about the evidence-based and person-centric PHV that fosters resilience in the face of health decline and the importance of resilience in promoting the well-being of older adults [17, 18, 20]. Therefore, we intend to systematically examine the effectiveness of PHV in enhancing resilience in addition to health, psychological and social-related outcomes among older adults. This review focused on the older adults living in the community and not institutionalised.

## Methods

This systematic review protocol was registered with the International Prospective Register of Systematic Reviews–PROSPERO (Registration number: CRD42022296919) and guided by the Preferred Reporting Items for Systematic Reviews and Meta Analysis (PRISMA) 2020 guideline [51, 52]. The PRISMA checklist is available in S1 File.

### Eligibility criteria

This review aimed to assess original research reporting the effectiveness of multidimensional PHV interventions. Studies were selected if there is a clear structure for the home visit interventions as preventive approach to assess and improve health and well-being of older adults living in the community. In addition to PHV, the studies will only be included in the review if resilience or any related concept is highlighted as the outcome of the study. The selection of studies follows the Population, Intervention, Comparator and Outcome (PICO) framework [53]. Table 1 presented the inclusion and exclusion criteria for this review.

**Table 1:** Inclusion and exclusion criteria.

Criteria	Inclusion criteria	Exclusion criteria
Population (P)	Older adults aged 60 years or above. Receive PHV or similar home visit intervention. Participants who were living in the community. Physically independent and mobile. However, participants who were partially immobile but needed help in performing IADL, pre-frail and frail also included as long as they live in the community and not fully disabled.	Older adults who were institutionalised or living at nursing/care home. Post-hospitalized or received specific home intervention for cure purposes and not for preventive approach. Home visit intervention for specific populations such as cancer patients or military veterans such as under the US Veteran Association (VA), Medicare beneficiaries etc.
Intervention (I)	PHV or any other type of home care provided by trained nurses or a multidisciplinary team consisting of a nurse, physical therapist, occupational therapist, social worker or other professionals such as a physician, dietitian, pharmacist or psychologist. In addition to regular home visits, intervention may include group meetings between participants and healthcare providers to increase health literacy and promote social participation.	Specific post-hospitalized or home care treatment / programme such as home rehabilitation programme, home-bound primary care programme (HBPC), special diet programme etc.
Comparator (C)	Usual care (for RCT studies)	
Outcomes (O)	Resilience or any related outcomes such as coping ability, adaptation, quality of life or well-being. Either health, psychology or social related outcomes or any combination of those.	No resilience or related outcomes. Single health/ psychological/ social outcome that not related to either resilience, quality of life or well-being.
Type of document	Quantitative primary research such as control trials, cross-sectional or quasi experimental studies. Earlier work(s) by the same authors including research protocols are also assessed and evaluated together with the selected study.	Qualitative research, conference papers, editorials and comments, case reports and preprints Review articles such as systematic review, scoping review, bibliometric analysis, narrative review etc. However, relevant citation from the review articles screened during this review are evaluated for inclusion.
Language	English only	
Time limit	No time limit	

### Search strategy

A literature search was conducted on nine online databases (PubMed, MEDLINE, CINAHL, Embase, Emcare, Web of Science (WoS), Scopus, PsycINFO and Cochrane Library) between March 15 and 31 March, 2022 using key search terms covering all four components of PICO combined with Boolean operator (AND and OR) and truncation (*). The keyword strings are as follows: (older adult* OR older people OR older person* OR aged OR elder* OR elder* people OR elder* person* OR senior citizen OR old* citizen) AND (preventive home visit* OR home visit* OR home care OR homecare OR house call) AND (resilien* OR coping OR adversit* OR bounce back OR positive adaptation OR protective factor*) AND (health status OR health outcome* OR disabilit* OR impair* OR frail* OR health related quality of life OR HRQoL OR quality of life OR unmet need* OR met need* OR need*.

We also searched grey literature from Google, Google Scholar and backward citations of the relevant documents related to this study. Google Scholar search was conducted with the keyword strings adjusted owing to the wordcount restriction by the search engine. Further updated searches were conducted on October 15, 2023 and again on April 10, 2024 for both the online databases and grey literature to assess and screen new records published after the previous search. The detail keyword search for online databases and grey literature is available in S2 File.

### Study selection

The search results from the online databases and grey literature were exported to EndNote 21 software (Clarivate Analytics, PA, USA) for duplicate removal. The data file (.txt) was then exported to the Rayyan.ai web application for systematic and effective data screening [54, 55]. Two independent reviewers (DBR and SM) conducted title and abstract screenings for all records and the screening results were then exported to Microsoft Excel for Mac (Version 16.66.1) for manual sorting and data management before continuing with full-text search. The same authors evaluated the inclusion of records into the systematic review after assessing the related full-text articles. Any discrepancies between them were resolved by the third reviewer (SS).

### Quality assessment

This review utilised the McMaster Critical Review Form for Quantitative Research for methodological quality evaluation [56]. This assessment tool is comprehensive to critically assess quantitative and qualitative methodology evidence, tailored to diverse study types and has good inter-rater reliability [57, 58]. This assessment tool was developed by an Evidence-based Research Group at McMaster University and is widely used among healthcare professionals to assess the quality of study design specifically but not limited to clinical and healthcare related studies. The assessment tool is comprehensive yet simple and can be easily leaned and utilised by a wide range of users from clinical experts to students as compared to a few other assessment tools that require technical and clinical expertise. The assessment consists of eight critical questions with three options to choose from (“yes (Y)”, “no (N)”, “not addressed (NA)” or “not applicable”). We modified the assessment form with two additional sub-items (ethics and participant informed consent) to enhance the compliance element in the methodological assessment. The total scores were calculated as percentages with records receiving a score less than 40% deemed low quality, 40.1% to 74.9% as fair, 75.0% to 79.99% as moderate and 80% and above as high [57, 58].

Furthermore, we analysed of the level of evidence in the studies involved by utilising the Australia National Health and Medical Research Council’s (NHMRC) Evidence Hierarchy Framework [59]. The evaluation involved five elements: 1) evidence-based; 2) consistency; 3) clinical impact; 4) generalizability; and 5) applicability. McMaster critical review form is used to examine the quality of records included in this review while NHMRC Evidence Hierarchy Framework provides clear hierarchical ranking of the evidence, which can be served as indicators for policy decision and recommendations. The integration of these two assessment tools facilitates more nuanced and comprehensive evaluation of the documents, ensuring a more robust methodology assessment.

### Outcome measures

The main aim of this study is to evaluate the effectiveness of PHV in enhancing resilience, in addition to its impact on health, psychological and social-related outcomes. Resilience can be recognised either as a distinct result or as a component of psychological outcomes. These two different aspects of resilience are considered unique due to the ongoing debates among resilience scholars from many disciplines regarding a unified definition of resilience [37, 42, 43, 60]. Health-related outcomes include functional capacity, self-rated health, mortality, morbidity, hospital or nursing home admission or readmission and any other relevant indicators and social outcomes encompass measures of social support, engagement, utilisation of social services or any other related outcomes associated with social services.

## Results

### Study selection

Search results from online databases yielded 5,621 records. A total of 2,056 duplicate entries were removed, followed by an exclusion of 3,656 records based on the specified exclusion criteria. We finally sought a total of 36 articles for retrieval. After excluding three protocol and trial registrations, 33 full-text research publications were selected for a comprehensive evaluation and only four articles met the inclusion criteria for the systematic review. Exclusion was due to a mismatched type of study such as descriptive studies without quantifiable data or articles describing PHV/resilience model design (n = 11), participants were from a different context such as those living in nursing homes or being post-hospitalised (n = 7), the studies did not report the effectiveness of PHV (n = 4), the interventions were only for specific participants instead of being designed as a multidimensional preventive approach (n = 5) and the studies only reported preliminary results (n = 2).

**Fig 1:**
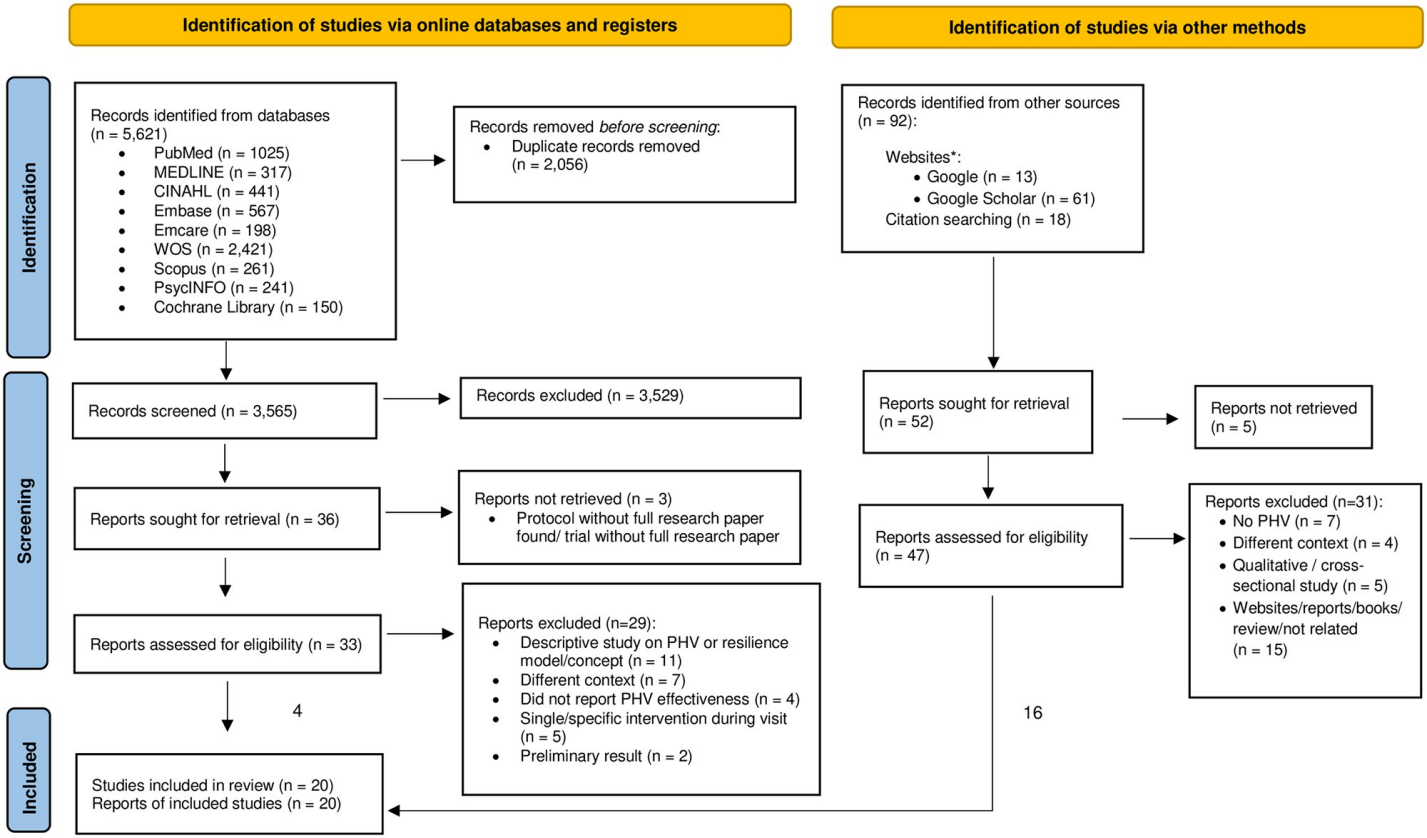
PRISMA flow diagram. From: Page MJ, McKenzie JE, Bossuyt PM, Boutron I, Hoffmann TC, Mulrow CD, Shamseer L, Tetzlaff JM, Akl EA, Brennan SE, Chou R. The PRISMA 2020 statement: An updated guideline for reporting systematic reviews. International Journal of Surgery. 2021 Apr 1; 88:105906. visit: http://www.prisma-statement.org/.

Keyword search using Google and Google Scholar were conducted with modification to fit the search engines' word length restrictions. The Google search produced 13 records whereas Google Scholar search yielded 61 records. Nevertheless, none of the records satisfy the specific inclusion criteria. In addition to screening publications from online databases, a backward citation search was conducted to identify potential articles to be included based on the relevant literature we assessed earlier, systematic reviews and meta-analysis on PHV. We identified a total of 18 records from previous literature sources. Out of a total of 92 records from grey literature, 47 records underwent comprehensive screening and 16 records were included from grey literature to make up the total of 20 articles analysed for this review. The screening procedure and findings for each stage following PRISMA guideline as illustrated in Fig 1.

### Methodological quality

Based on the McMaster critical review assessment, level of evidence and critical appraisal score were calculated and tabulated (Table 2). 17 studies were classified as high quality with a total score of 80% or more. Additionally, three studies were categorised as having moderate quality, scoring between 75.0 and 79.99%. The majority of the studies were designed with a robust methodology framework, including clearly defined purposes (item 1), supported by relevant literature (item 2), employing appropriate measurement instruments and analysis techniques (item 5, 7a &7b) and providing a comprehensive explanation of the interventions (6a). However, the majority of the studies did not comply with two components of the assessment, namely avoiding co-intervention (item 6b) and reporting clinical importance (7c). In most developed countries, the controlled trials were challenged by counter-intervention. Despite not receiving PHV intervention, the control group received adequate healthcare and social care from the providers, which effectively addressed their health and psychosocial needs, hence the outcome measures among the control group were affected by the external services they acquired. In terms of clinical application, nine out of twenty studies failed to adequately elucidate the clinical significance of their findings, including any potential adverse health or psychosocial implications for the examined population or older individuals in general.

**Table 2:** Level of evidence and critical appraisal scores.

Study/McMaster review component	1	2	3	4a	4b	4c	4d	5a	5b	6a	6b	6c	7a	7b	7c	7d	8	Total	Quality assessment
1. van Rossum et al. (1993) [61]	Y	Y	RCT (2-armed)	Y	Y	NA	Y	Y	Y	Y	Y	N	Y	Y	Y	Y	Y	14/16	High
2. Stuck et al. (1995) [62]	Y	Y	RCT (2-armed)	Y	Y	Y	Y	Y	Y	Y	Y	N	Y	Y	N	Y	Y	14/16	High
3. Stuck et al. (2000) [63]	Y	Y	RCT (2-armed)	Y	Y	Y	Y	Y	N	Y	Y	NA	Y	Y	Y	Y	Y	14/16	High
4. van Hasstregt et al. (2000) [64]	Y	Y	RCT (2-armed)	Y	Y	Y	Y	Y	Y	Y	NA	NA	Y	Y	Y	Y	Y	13/16	High
5. Hebert et al. (2001) [65]	Y	Y	RCT (2-armed)	Y	Y	NA	Y	Y	Y	Y	N	N	Y	Y	Y	Y	Y	13/16	High
6. Yamada & Ikegami (2003) [66]	Y	Y	RCT (2-armed)	Y	Y	NA	Y	Y	Y	Y	N	Y	Y	Y	NA	Y	Y	13/16	High
7. Markle-Reid et al. (2006) [67]	Y	Y	RCT (2-armed)	Y	Y	Y	Y	Y	Y	Y	N	Y	Y	Y	Y	Y	Y	15/16	High
8. Gitlin et al. (2009) [68]	Y	Y	RCT (2-armed)	Y	Y	NA	Y	Y	Y	Y	Y	Y	Y	Y	Y	Y	Y	15/16	High
9. Ploeg et al. (2010) [69]	Y	Y	RCT (2-armed)	Y	Y	Y	Y	Y	Y	Y	Y	N	Y	Y	N	Y	Y	15/16	High
10. van Hout et al. (2010) [70]	Y	Y	RCT (2-armed)	Y	Y	Y	Y	Y	Y	Y	Y	N	Y	Y	N	Y	Y	14/16	High
11. Behm et al. (2014) [71]	Y	Y	RCT (3-armed)	Y	Y	Y	Y	Y	Y	Y	Y	Y	Y	Y	Y	Y	Y	16/16	High
12. Godwin et al. (2016) [72]	Y	Y	RCT (2-armed)	Y	Y	Y	Y	Y	Y	Y	NA	NA	Y	Y	N	Y	N	12/16	Moderate
13. Kono et al. (2016) [73]	Y	Y	RCT (2-armed)	Y	Y	Y	Y	Y	Y	Y	NA	NA	Y	Y	Y	N	Y	13/16	High
14. Sherman et al. (2016) [74]	Y	Y	CCT (2-armed)	Y	Y	Y	Y	Y	Y	Y	NA	NA	Y	Y	Y	Y	Y	14/16	High
15. Arola et al. (2018) [75]	Y	Y	RCT (2-armed)	Y	Y	Y	Y	Y	N	Y	Y	Y	Y	Y	NA	Y	Y	14/16	High
16. van Lieshout et al. (2018) [76]	Y	Y	RCT (2-armed)	Y	Y	Y	Y	Y	Y	Y	Y	N	Y	Y	NA	Y	Y	14/16	High
17. Liimatta et al. (2019) [77]	Y	Y	RCT (2-armed)	Y	Y	Y	Y	Y	Y	Y	Y	Y	Y	Y	Y	Y	Y	16/16	High
18. Szanton et al. (2019) [78]	Y	Y	RCT (2-armed)	Y	Y	Y	Y	Y	Y	Y	Y	Y	Y	Y	Y	Y	Y	16/16	High
19. Hoedemakers et al. (2022) [79]	Y	Y	Quasi-experimental	Y	N	Y	Y	Y	Y	Y	NA	NA	Y	Y	N	Y	Y	12/16	Moderate
20. Bloternberg et al. (2023) [80]	Y	Y	Longitudinal study	N	NA	Y	Y	Y	Y	Y			Y	Y	N	Y	Y	11/14	Moderate

RCT: Randomised controlled trial; CCT: Controlled clinical trial; Y = yes, N = No, NA = not addressed, highlighted in black = not applicable.

McMaster critical review items [56]:

1.Was the purpose stated clearly?; 2. Was relevant background literature reviewed?; 3. What is the study design?; 4a. Was the sample described in detail?; 4b. Was sample size justified?; 4c. Ethics procedure explained?; 4d. Inform consent obtained?; 5a. Were the outcome measures reliable?; 5b. Were the outcome measures valid?; 6a. Intervention was described in detail?; 6b. Contamination was avoided?; 6c. Cointervention was avoided?; 7a. Results were reported in terms of statistical significance?; 7b. Were the analysis method(s) appropriate?; 7c. Clinical importance was reported?; 7d. Drop-outs were reported?; and 8. Conclusions were appropriate given study methods and results?.

Quality assessment: Low: < 40%; Fair: 40.1% to 74.9%; Moderate = 75.0% to 79.99%; High: 80% and above

In addition, we utilised NHMRC Evidence Hierarchy Framework assessment (Table 3) to further evaluate the quality of evidence for the studies analysed in this review. 17 out of 20 studies were RCTs and were considered to have a high degree of methodological quality (level II). One study was a clinical controlled trial, another was quasi-experimental research and both were grouped as level III-2. There was one longitudinal study and ranked as level 4. The available evidence from the research included in this review which related to consistency, generalisability, and the overall grade for recommendation was satisfactory while the clinical impact derived from these studies was found lacking and graded as “poor”.

**Table 3:** NHMRC body of evidence framework.

Component	Grade	Comments
Evidence base	B–Good One or two level II studies with low risks of bias/ level III and IV studies with low risk of bias	Quantity: 20 studies (18 RCTs, 1 quasi-experimental, 1 longitudinal) Participants: 8,035 community-dwelling older adults with approximately two-third were independent and mobile and approximately one-third were pre-frail and frail. 4 studies involving sub-population analysis. Evidence hierarchy: Level II: 18 studies Level III-2: 1 study Level 4: 1 study
Consistency	C–satisfactory Some inconsistency reflecting genuine uncertainty around clinical question	Multiple study designs: RCTs, quasi-experimental and longitudinal studies All studies reported statistical significance. All studies that use PHV as an intervention are heterogeneous in terms of: Participants selection Duration and follow-ups (from 5 to 48 months) interveners (only a nurse or multidisciplinary team) PHV approaches Assessment tools and outcomes
Clinical impact	D–Poor Slight or restricted	While all studies reported statistical significance, only 11 studies (55%) reported clinical significance. Most studies did not discuss the effect size on the results obtained.
Generalisability	C–satisfactory population/s studied in the body of evidence are similar to the target population for the guideline	Population of the studies was mainly healthy older adults living in the Western countries. Studies were conducted in 8 countries with different healthcare contexts. Out of 20 studies, only two represented the East (Japan) while the rest were from European countries (Sweden (3), the Netherland (5), Switzerland (1), Finland (1), Germany (1)) and the rest were from Canada (4) and the United States (3). All studies excluded participants who were physically and cognitively impaired.
Grade of recommendations	C–Satisfactory Body of evidence provides some support for recommendation(s) but care should be taken in its application	Overall, most studies had low risks of bias, while two studies had moderate risks of bias. While the evidence base of most of the studies was good, outcomes measuring the effectiveness of PHV were inconsistent due to co-intervention issues in several studies in which participants may have received good healthcare services on top of the intervention throughout the study period. Heterogeneity in PHV implementation, especially with diverse assessment tools, warrants uniformity in developing a PHV framework. The focus on resilience as an outcome for PHV is lacking (except for one study). Hence, a holistic PHV framework with multidomain interventions including resilience as a psychosocial indicator is essential.

### Study characteristics

Over the past thirty years, more than 1,000 studies investigating the effectiveness of PHV among older adults have been conducted based on WOS, Scopus and Google Scholar searches. The results of keyword search for this review is specifically focusing on the effectiveness of PHV on resilience on top of health, psychological and social related outcomes. The research on this topic has been emerging since 1980s and the trend has been escalating since 2010. The most recent study on this topic was published in 2023 [79]. A total of 20 studies that met the specified criteria were primarily carried out in European nations. Specifically, five studies originated from the Netherlands [61, 64, 76, 79, 70], three from Sweden [71, 74, 75] and one each from Finland [77], Switzerland [63] and Germany [80]. The remaining trials were conducted in Canada [65, 67, 69, 72], the United States [62, 68, 78] and Japan [66, 73].

### Participant characteristics

This review involved a total of 8,035 participants, who the mean aged ranged from 74.0 to 84.4 years. More than half of the studies reported a mean age of 75 years or more, whereas three studies reported age ranges and the corresponding mean age [61, 67, 74]. All study participants were predominantly women, ranging from 51% to 87.2% of participants. The largest sample comprised 791 older adults who were at risk of being admitted to a nursing home [63]. The smallest sample included 75 healthy older adults living in Emlichheim, a region in North-western Germany [80].

There were four distinct groups of participants among the studies in this review. First, the review included a majority of study participants among healthy and independent older adults population who did not require on home help services and did not have any physical or cognitive impairment [61, 62, 67, 70–72, 74, 75, 77, 80]. The second group consisted of the individuals who experienced difficulties with performing Activities of Daily Living (ADLs) or Instrumental Activities of Daily Living (IADL) [68, 78], limited mobility [19, 64] or at risk of functional decline [65, 69]. The third group consisted of older adults who were classified either frail [15, 66, 67, 70, 79] or pre-frail or less frail [73, 76]. Finally, one study was conducted to examine two distinct groups, which is high and low risks of being admitted to a nursing home [63].

### Type of interventions

The PHV intervention exhibits significant heterogeneity in the terminology used to define the intervention and the mechanisms employed for its execution. According to the data presented in this review, the intervention referred to as "preventive home visit” mentioned in four studies [66, 71, 74, 77]. In contrast [61] used the more general term “home visit”. Other terms used were “home-based nursing intervention programme” [72, 75] and “nursing health intervention programme” [67]. The studies that emphasised multidimensional intervention were referred to as “home-based multidisciplinary programme” [78], “multidimensional preventive programme” [65] and “proactive multicomponent intervention programme” [76]. In addition, certain studies employed specific phrases to describe their intervention such as “in-home CGA” or “multidimensional CGA assessment” [62, 63], “home-based occupational and physical therapy intervention” [68] and “person-centric integrated care programme” [79].

The duration of the intervention ranged from five to 48 months. It is administered either by dedicated nurses or multidisciplinary professionals comprising public health nurses (PHNs), district nurses (DNs), registered nurses (RNs), primary care nurse specialists (PCNSs), psychosocial nurses, nurse practitioners as well as other professionals such as physiotherapists (PTs), occupational therapists (OTs), dietitians, social workers (SWs), care managers (CMs) or research assistants (RAs).

The summary of study characteristics including the type of interventions is presented in Table 4.

**Table 4:** Study characteristics.

No.	Author (Year) Country	Study design	Sample size (I/C/*SM)	Research Setting	Participant Characteristics	Age criteria, MA (SD)/range (I/C)	Intervention (Intervener)	Intervention period (Baseline and Follow-up)	Comparator	Outcome (s)
1.	vanRossum et al. (1993) [61] The Netherlands	2-armed RCT	580 (292/288)	Weert (South Netherland)	Community-dwelling, not receiving home nursing care Female (I/C) (%): 58/57 Living alone (I/C) (%): 39/39	75+ years Range: 75–79 years (I/C) (%): 72/73 80–84 years (I/C) (%): 28/27	Home visit (PHN)	36 months (4 times a year)	Usual care	Primary Functional state Self-rated health Mortality Well-being Mental state Secondary Community and institutional care utilisation Health service cost
2.	Stuck et al. (1995) [62] USA	2-armed RCT	414 (215/199)	Santa Monica	Independent living without severe physical and cognitive impairment Female (I/C) (%): 69/71 Living alone (I/C) (%): 65/63 Completed high school (I/C) (%): 80/76	75+ years MA (SD) I/C: 81(3.9)/81.4(4.2)	In-home visit with annual CGA evaluation (Gerontologic nurse with involvement of geriatrician or physician if necessary)	36 months (0,12,24,36 month)	Usual care	Primary Prevention of disability Prevention of nursing home and hospital admission Secondary Community services utilisation Visit to physician Healthcare/intervention service cost
3.	Stuck et al. (2000) [63] Switzerland	Stratified 2-armed RCT	791 (264/527) Low risk: 148/296 High risk: 116/231	Bern	Older adults with low and high risks of nursing home admission (based on 6 baseline risks for nursing home admission) Women (I/C) (%): 77/71 Living alone (I/C) (%): 57/54 Married (I/C) (%): 35/39 Complete 9^th^ grade or less (I/C) (%): 75/73	75+ years, whites MA(SD) I/C: 82(4.7)/81.5(4.5)	In-home visit with annual CGA and follow-ups (Visit by 3 PHN based on 3 different zip code area with involvement of intradisciplinary team (PT, OT, dietitian, SW) if necessary)	36 months (0, 24-month in-home visit follow-up, 36-month telephone follow-up)	Usual care	Primary Assistance in performing ADL and IADL Nursing home admission Secondary Health status Mortality Healthcare and ambulatory utilisation Satisfaction with the programme Healthcare and ambulatory cost
4.	van Haastregt et al. (2000) [64] The Netherlands *Home visit protocol*: *Stalenhoef et al*. *(1998)*	2-armed RCT	316 (159/157)	Hoensbroek, the Netherlands	Living in the community, have reported fall for the past six months or have moderate mobility challenges. Women (I/C) (%): 65/67 Below average income (I/C) (%): 62/66 Living alone (I/C) (%): 50/50 Elementary school or less (I/C) (%): 46/56	70+ years MA (SD) (I/C): 77.2 (5.1)/77.2(5.0)	Five home visits in a year, around 50 minutes each session. Visits include health and psychosocial screening, fall risks assessment and home hazard assessment including behavioural factors that potentially influencing falls and mobility. (community nurse)	18 months (0, 12,18)	Usual care	Primary Fall Mobility impairment Secondary Physical function Perceived health Perceived gait problems Daily activities Fear of fall Mental health Loneliness Social functioning
5.	Hebert et al. (2001) [65] Canada *Detail on study design*: *Hebert et al*. *(1996)*	2-armed RCT	503 (250/253)	Sherbrooke City, Quebec	Community-dwelling with risk of functional decline Women (I/C) (%): 64/64.4 Married (I/C) (%): 44.4/46.6	75+ years MA(SD) I/C: 80.2(4.2)/80.3(4.5)	Nurse-led multidimensional preventive programme with health screening analysed by GP, a monthly follow-up phone call (Trained nurse with reference to GP if necessary)	12 months (0,12 month)	Usual care	Primary 1. Functional decline Disability Mortality Institutionalisation Secondary General health Functional autonomy Well-being Healthcare services utilisation Perceived social support and integration
6.	Yamada & Ikegami (2003) [66] Japan	2-armed RCT	368 (184/184)	Sapporo and Takahata	Frail community-dwelling, independent in ADL but dependent in IADL Sub-group: participants who perceived their health status as poor at baseline and those who complied with the advice given by the nurses during the visit Women full population (%): 63.3% Women (I/C) (%): 64.1/62.5 Living alone (I/C) (%): 9.8/8.7	65+ years MA (SD) full population): 78.7(7.1) MA(SD) I/C: 78.7(7.0)/78.7(7.2)	PHV Multidomain health assessment using Minimum Data Set-Home Care (MDS-HC) 2.0 and care plan (PHN)	18 months (0,18)	Usual care	Primary HRQoL Secondary Mobility Self-care Usual activities Pain/discomfort Anxiety/ depression Health behaviour
7.	Markle-Reid et al. (2006) [67] Canada *Detail intervention in unpublished thesis*: *Markle-Reid (2002)*	2-armed RCT	288 (144/144)	Participants of home care programme in Ontario, Canada	Frail older adults who received home care (proactive nursing health promotion intervention) from the Community Care Access Centre (CCAC) programme, a publicly funded home care service. Female (I/C) (%): 77.5/76.2 High School education (I/C) (%): 47.1/45.1 Income below $40,000 full population (%): 87.2 Income below $40,000 (I/C) (%): 90/84.4	75+ years Range (full population): 75–85 years (%): 69.4 86+ years (%): 30.6 Range (I/C): 75–85 years (%): 75/63.9 86+ years (%): 25/36.1	Proactive nursing health promotion interventions with home care services consist of case management, occupational therapy and physiotherapy, speech therapy and social work. (RN, RA and CM (if necessary)	6 months (0,6 months)	Home care (nursing services on demand)	Primary Not specified General outcomes HRQoL Functional health status Mental health Mental health–depression Health and social care utilisation Coping style Social engagement rate Perceived social support Health and social service cost
8.	Gitlin et al. (2009) [68] USA *Earlier studies*: *Gitlin et al*. *(2006a)*, *Gitlin et al*. *(2006b)*, *Gitlin et al*. *(2008)*	2-armed RCT	319 (160/159)	Philadelphia	Having difficulties in performing daily activities and cognitively intact. Participants were categorised by mortality risk level (Lee’s et. al prognostic item) Women (full population): 81.8% Living alone (full population): (61.8%) White (53%) African America (46%)	70+ years MA (SD) full population: 79 (5.9)	Home-based occupational and physical therapy intervention: Advancing Better Living for Elders (ABLE) with two phases: 1) targeting physical health, physical therapy, home modifications and safety assessment 2) Maintenance phase: brief phone call follow-up and another home visit in the end of session (OT, PT)	48 months (12, 24, 36, 48 month)	Usual care and home safety education	Single outcome Survivorship (mortality)–through control support mechanism during ABLE intervention which involve health professional visits, home safety and referral functions and social interaction between the visitors and beneficiaries
9.	Ploeg et al. (2010) [69] *Home care protocol*: *Morris JB et al*. *(2002)* Canada	RCT (2-armed)	719 (361/358)	Hamilton, Ontario	Older adults with high risk of functional decline, patients from 35 family physicians in 5 primary care networks in Hamilton, Ontario. Subgroup analysis for high and low risk of functional decline. Women (I/C) (%): 52/54 Living alone (I/C) (%): 33/35	75+ years MA (SD) (I/C): (81.0 (4.1)/81.3 (4.4))	Comprehensive health assessment for home care and follow up. Visit Includes health promotion advice and recommendation to community health and social support services. (home care nurse)	12 months (6,12)	Usual care	Primary Quality adjusted life years (QALYs) Secondary Self-rated health Functional status ADL IADL Cost of healthcare utilisation Prescription drugs cost Health and social care cost Nursing cost
10.	van Hout et al. (2010) [70] *Assessment of health risk*: *(Hebrew Senior Life (2005) and Landi et al*. *(2000)* The Netherland	RCT (2-armed)	651 (331/320)	Netherland (no specific location mentioned)	Frail older adults who were the patients from 33 Primary care practices and 55 Primary Care Physician (PCPs) Women (I/C) (%): 72.2/68.8 Living alone (I/C) (%): 54.7/55.0 No or low education (I/C) (%): 73.1/71.9	75+ years MA (SD) (I/C): (81.3(3.9)/81.5(4.3))	PHV 4 times a year with comprehensive home assessment consist of 7 components which includes health risk and care needs using Resident Assessment Instrument–Home Care (RAI-HC) assessment, person-centric care plan intervention consultation to physician and follow-ups visits if necessary (community nurse)	18 months (0,6,18)	Usual care	Primary Functional health Disability in IADL Secondary Mortality Institutionalisation Acute hospital admission Mental health Cognitive impairment Depression and anxiety Loneliness
11.	Behm et al. (2014) [71] Sweden *Study protocol*: *Dahlin-Ivanoff et al*. *(2010)* *Earlier study on 3-month short term result*: *Gustafsson et al*. *(2012)*	3-armed RCT	459 (174/114/171*)	2 urban cities in Gothenburg	Community-dwelling, independent of ADL and cognitively intact Female (PHV/SM/C) (%): 64/66/61 Living alone (PHV/SM/C) (%): 57/60/48 Self-rated health (excellent/very good/good) (%): 80/83/79	80+ years MA (range) (PHV/SM/C): 86(80–94)/85(80–94)/86(80–97)	PHV which consist of guide on social and health care services available, health and fall risks assessment and home hazard and modification advice. Four weekly senior meeting with a small group discussion on ageing concept, consequences and responses. (RN, PT, OT, SW)	24 months (0,12,24) PHV lasted for 1 ½ to 2 hours SM lasted for 2 hours including coffee break	Usual care	Primary Frailty Secondary Morbidity Health symptoms Self-rated health Satisfaction with physical and psychological health
12.	Godwin et al. (2016) [72] Canada	2-armed RCT	236 (121/115)	St. John’s, Newfoundland	Community-dwelling, independent, cognitive intact, not receiving home care services. Women (full population) (%): 66.5% Women (I/C) (%): 62/71.3 Post-secondary education (full population) (%): 43.6 Post-secondary education (I/C) (%): 52.1/34.8 Live with a partner (I/C) (%): 42.1/32.2	80+ years MA(SD) full population: 85.5(4.1) MA (SD) (I/C): 85.3(4.5)/85.7(3.6)	Home-based nursing care intervention programme with Personalized ElderCare Plan to meet individual’s need. Up to eight visits in 1-year period (PCNS and RA)	12 months (0,6,12)	Usual care	Primary Symptomology HRQoL Satisfaction with care Secondary Healthcare utilisation Visit to ED Visit to physician Utilisation of diagnostic services Anxiety/ depression Social functioning
13.	Kono et al. (2016) [73] Japan *An updated RCT of Kono et al*. *(2012) and Kono et al*. *(2013)* *Research protocol*: *Kono et al*. *(2014)*	2-armed RCT	360 (179/ 181)	Suburban municipalities in Osaka, Japan:	Less frail and frail older adults under Japanese Long-term Care Insurance (LTCI) system Women (I/C) (%): 75.4/72.4 Less frail (support level 1) (I/C) (%): 49.7/49.2 More frail (support level 2) (I/C) (%): 50.3/50.8 Living alone (I/C) (%): 41.3/46.9	65+ years MA (SD) (I/C): 79.2(6.0) / 79.2 (6.3)	PHV routine for every three months based on structured multidimensional care needs assessment (physical and mental health, activities and participation) followed by comprehensive recommendations. (Community care nurses, SWs, CMs)	36 months (Every 3 months for 24 months, up to 36 months for care need level and LTC service utilisation)	Usual care with unstructured visit under the LTCI system	Primary Functional ability (at 12 and 24 month) ADL/IADL Fall Hospitalisation Depression (including question on the death of family members) Cognitive Social activities satisfaction Self-efficacy for health promotion Care need level (at 12, 24, 36 month) Secondary LCT service utilisation (over the past 36 months) Cost of LCT utilisation
14.	Sherman et al. (2016) [74] Sweden	Cluster-controlled Trial	438 (176/262)	Stockholm	Older adults from 16 healthcare centre (HCC) registries, living at home, no dementia or serious illness such as stroke. Women (%) (I/C): 53/52 Living alone (%) (I/C): 40/32 Education–Elementary School (%) (I/C): 45/50	75+ years MA (SD): not stated	PHV consist of health dialogue, assessment of health, planning, diagnosis of health needs, nursing intervention and evaluation of nursing care. (DN, supports from researchers, doctors and staff at HCC (if necessary)	0, 12 months (12 –in between 3 to 11 months, median 6 months)	Usual care	Primary Not specified General Self-rated health: Health index–Energy, mood, fatigue, vertigo, bowel function, well-being. Loneliness General health Health problems Use of medication Knowledge about healthcare services Experience and usefulness of PHV
15.	Arola et al. (2018) [75] Sweden *Study protocol and baseline assessment*: *Gustafsson et al*. *(2015)*	2-armed RCT	131 (56/75)	Two suburban area of Sweden	Immigrant from Finland and Balkan Peninsula, independent and cognitively intact Male (%) (I/C): 49/52 Living alone (%) (I/C): 51/43 Live in Sweden more than 21 years (%) (I/C): 91/63	70+ years MA (SD full population): 74.1 (3.4) MA (SD) (I/C): 74.0(3.4)/74.2(3.4)	Home-based nursing intervention programme consist of 4 weeks SMs and one PHV follow-ups. The intervention was part of Promoting Aging Migrants’ Capability (PAMC) project. (OT, PT, RN and SW or trained RA during baseline then RA at 6^th^ & 12^th^ months follow-up)	12 months (0,6,12)	Usual care	Specifically focus on Sense of Coherence (SoC) based on three dimensions: Comprehensibility Manageability Meaningfulness Home-based multidomain nursing intervention targeting functional ability (ADL), physical performances, FoF, symptoms, life satisfaction, depression and social interaction.
16.	van Lieshout et al. (2018) [76] The Netherlands	2-armed RCT	281 (139/142)	semi-rural community, central Netherlands	Pre-frail community-dwelling, independent, mobile and cognitively intact Female (%) (I/C): 59/51.4 married or cohabiting (%) (I/C): 56.8/66.2 Pre-frail (%) (I/C):58.3/60.6 Frail (%) (I/C):41.7/39.4	65+ years MA (SD) full population: 74(7.2) MA (SD) (I/C): 73.3(6.7)/74.7(7.6)	23-week multicomponent programme: Supporting Proactive Lifestyle Intervention in Frailty and Disability (SPRY)—individual and group-based assessment (PT, psychosocial nurse, dietician, professional in welfare)	12 months (0,6,12)	Unclear	Primary Functional ability (ADL) Secondary Functional ability (IADL) Functional capacity Physical fitness Walking speed Medication Mobility Nutrition Healthcare utilisation QoL Depression Loneliness
17.	Liimatta et al. (2019) [77] Finland *Method and preliminary findings*: *Liimatta et al*. *(2017)*	2-armed RCT	422 (211/211)	Hyvinkaa town municipality	Independent community-dwelling older adults, not receiving home help or nursing services Women (I/C) (%): 65/65 Black (I/C) (%): 89.9/82.9 Cohabiting (I/C) (%): 52/51 Less than 9 years education (I/C) (%): 47/50	75+ years MA (SD) (I/C): 80.8 (4.3)/81.3(4.3)	PHV and intervention in between 6 to 9 months by multidisciplinary team with 30 to 90 minutes for each visit (RN, PT, SW)	24 months (0,12,24)	Usual care	Primary Mortality Secondary Health-related QOL
18.	Szanton et al. (2019) [78] USA *Study design*: *Szanton et al*. *(2014)*	2-armed RCT	300 (152/148)	Baltimore, Maryland	Low income, cognitively intact older adults, self-reported difficulty (at least 1 ADL or 2 IADL) Women (I/C) (%): 87.2/87.5 Black (I/C) (%): 89.9/82.9 Living alone (I/C) (%): 47.3/52.6 Education more than 12 years (I/C) (%): 63.3/71.0	65+ years MA (SD) full population: 75.7 (7.6) MA (SD) (I/C): 75.7 (7.6)/75.4(7.4)	Home-based multidisciplinary programme: Community Aging in Place–Advancing Better Living for Elders (CAPABLE)–consists of 10 sessions over 5 months and home-based multidisciplinary assessment with home modifiers (RN, OT and RA)	12 months (0,5,12)	10 home visits (1 hour each) over 5 months	Primary 1. Functional ability (ADL and IADL) at 5 months Secondary 2. Functional ability (ADL and IADL) at 12 months 3. Perceived benefit of PHV
19.	Hoedemakers et al. (2022) [79] The Netherlands	prospective quasi-experimental by using Multi-Criteria Decision Analyses (MCDA)	384 (222/162)	CCFE participants	Frail community-dwelling older adults among Care Chain Frail Elderly (CCFE) participants Women (I/C) (%): 63.5/64.2 Living alone (I/C) (%): 54.1/61.1 Low education (I/C) (%): 70.3/70.4	80+ years MA (SD) (I/C): 83.4/84.8 (2.2)	Home visit with care support and personalized individual care plan by multidisciplinary team (DS, GP, nurse practitioners)	12 months (0,6,12)	Usual care received by CCFE participant who have not yet started the programme	General Physical function Burden of medication Psychological well-being Enjoyment of life Resilience Autonomy Experience of care Social relationships Social participation Healthcare, medication, social care and informal care cost Bundle payment of chronic care programme
20.	Blotenberg et al. (2023) [80] Germany	Longitudinal study	75	Emlichheim (North- western, Germany)	Older adults in the rural area of German (living in the join municipality of Emlichheim), cognitively and physically intact and not receiving benefits from long-term care insurance. Female (%): 64 Living with partners (%): 61.3 Elementary school /school leaving certification (%): 52%	65+ years MA (SD): not stated Range (%): 65–69: 27.4 70–74: 32.3 75–79: 32.3 80–85: 8.0	PHV with consultations involving 4 visits (T1 –T4). Personalized preventive plan was developed using the Standardized Assessment of Elderly People in Primary Care in Europe with the module mobility (STEP-m) (professional nurse)	10 months (0,1,3,6,10)	Not applicable	General Health related QoL Physical health Psychological health Mental health Social functioning 6 Specific cases were presented to show external influence on the outcome of PHV effectiveness.

I: Intervention

C: Control

SM: Senior meeting @ Senior group meeting

MA: Mean age

SD: Standard deviation

RCT: Randomised controlled trial

PHV: Preventive home visit

CGA: Comprehensive Geriatric Assessment

RCT: Randomised Controlled Trial

ADL: Activities of Daily Living

IADL: Instrumental Activities of Daily Living

FoF: Fear of fall

SRH: Self-rated health

QoL: Quality of Life

HRQoL: Health-related QoL

HCC: Healthcare centre

ED: Emergency department

DN: District nurse

RN: Registered nurse

PHN: Public health nurse

GP: General practitioners

OT: Occupational therapist

PT: Physiotherapist

PCNS: Primary care nurse specialist

CM: Care manager

SW: Social worker

RA: Research assistant

### Outcome measures

This review specifically examines the effectiveness of PHV on resilience as well as health, psychology and social outcomes. All studies involved the assessment of health conditions of either at baseline or during PHV follow-up period. Regarding the outcome measures, all studies except [75] assessed health related outcomes, sixteen studies measured psychological outcomes while only seven studies particularly focused on social-related outcomes (Table 5).

**Table 5:** Outcomes measures and main findings.

No.	Author/Year	Outcome measures (tools used) and findings						Conclusion
		Health		Psychology		Social		
1.	vanRossum et al. (1993)	Primary Functional state (5 questions questionnaire) SRH (questionnaire: 0-poor health to 10-excellent health) Mortality (follow up during visit) Others Institutional care utilisation (Interview with the community or care centres) *Health service cost (calculation of average total cost per service)*	⊗ ⊗ ⊗ ⊗ ↑(-)	Primary Well-being Mental state Depressive complaints (Zung’s self-rating Depression Scale) Memory disturbance (abbreviated mental test) Loneliness (Rasch-type Loneliness Scale and and Van Enn meet-instrument)	⊗ ⊗	Others Community care utilisation (Interview with the community or care centres)	↑(+)	PHV was not effective for the general population but could be effective for subpopulation participants with poor health
2.	Stuck et al. (1995)	Primary Prevention of disability assistance in performing ADL (interview) assistance in performing and IADL (Lawton & Brody scale) Prevention of hospital admission for acute care (self-declared & medical records) Others Prevention of permanent nursing home admission (self-declared & medical records) Prevention of short-term nursing home admission (self-declared & medical records) Visit to physicians (Medicare claims and data form local health maintenance organizations) *Cost*, *disability-free life years (estimation of disability-free years or preventing one day of permanent stay in nursing home)*	↓ (+)* ⊗ ⊗ ↓(+)* ⊗ ⊗ ↓(+)*			Others Community services utilisation (interview via phone call)	⊗	In-home comprehensive geriatric assessment was effective in delaying disability and can partially reduce permanent nursing home admission among the participants
3.	Stuck et al. (2000)	Primary Need for assistance in performing ADL (interview) Need for assistance in performing IADL (Lawton & Brody scale) entire sample among low baseline risk participants among high baseline risk participants Nursing home admission (phone call and verification from health insurance registry) entire sample among low baseline risk participants among high baseline risk participants Others For participants in low baseline risk 1. Health status (GDS-15, MMSE, 28-point gait and balance examination, COOP) 2. Number of medications 3. Influenza vaccines status 4. Mortality 5. Healthcare utilisation 6. Visit to primary care 7. Visit to specialist care (no 2–7, data were form interview and insurance data) No effect for all other outcomes among the high baseline risk 8. *Total cost saving*, *low risk group only (tax information)*	⊗ ↓(+)* ↓(+)* ⊗ ⊗ ↓(+)* ↑(-)* ⊗ ⊗ ↑(-)* ⊗ ⊗ ↑(+)* ⊗ ↓(+)	Others Satisfaction with the programme	⊗			Intervention can reduce disabilities among participants with low functional impairment risks but not for among with high risks. The effect was highly influenced by the performance of the interveners
4.	van Haastregt et al. (2000)	Primary Fall (mobility control scale and mobility range scale–short version) Mobility impairment Secondary Physical complaints (questionnaire) Perceived health (RAND-36) Perceived gait problems (5 -Likert scale questionnaire) Daily activity (13 item Frenchay activities index) At 12 months At 18 months Fear of fall (Falls efficacy scale)	⊗ ⊗ ⊗ ⊗ ⊗ ↓(-)* ⊗ ↓(+)*	Mental health (RAND-36) Loneliness (six-point Likert scale questionnaire)	⊗	Secondary Social functioning (adjusted version of item 4 and 5 of the social activities battery)	⊗	The PHV intervention had no effects on fall and mobility among the IG and CG. Falls and mobility impairment remain as serious problem among older adults in the context of Dutch healthcare setting hence alternative strategies should be developed and tested in various healthcare settings.
5.	Herbert et al. (2001)	Primary Functional decline Disability (SMAF) Mortality (visit/telephone call) Institutionalisation (questionnaire on total number of admissions to nursing home or long-term care hospital) Secondary General health (Dupuy General Well-being) Functional autonomy (SMAF) Health care services utilisation	⊗ ⊗ ⊗ ⊗ ⊗ ⊗	Secondary 2. Well-being (Dupuy General Well-being) anxiety depression, self-control vitality	⊗	Secondary 1. Perceived Social support and integration	⊗	The efficacy of multi-professional programme for preventing functional decline could not be demonstrated in this study.
6.	Yamada & Ikegami (2003)	Primary 1. Health Related QoL (EuroQoL 5 Dimension (EQ-5D)) Secondary Mobility (item 1—EQ-5D) Self-care (item 2—EQ-5D) Usual activities (item 3—EQ-5D) Pain/discomfort (item 4—EQ-5D)	⊗ ⊗ ⊗ ⊗ ⊗	Secondary Anxiety/depression (item 5—EQ-5D) Health behaviour (data from annual Ministry of Health & Welfare survey)	⊗ ⊕^a^			PHV was ineffective for the whole studied participants who were dependent in IADLs but independent in ADLs but effective for the IG among subpopulations with poor SRH and subpopulations who comply with PHN’s advice.
7.	Markle-Reid et al. (2006)	General outcomes HRQoL (SF-36) functional health mental health component *Health and social service cost (Health and Social Service Utilisation Inventory (Browne et al*. *2001a))*	↑(+) ↑(+)* ⊕^b^	General outcomes HRQoL Mental health, present of depression (CCAC record and Short Portable Mental Status Questionnaire) Coping style (Coping Questionnaire (Moos et al. 1985)	↓(+)* ⊗	General outcomes Social engagement rate (Nursing agency record) Perceived social support (Personal Resource Questionnaire 85 (Weinert & Brandt 1987))	87.4% ↑(+)*	Home-based intervention without any additional healthcare costs were effective in enhancing QoL among frail older adults with chronic health condition.
8.	Gitlin et al. (2009)	Primary 1. Survivorship–Mortality (data form National Death Index record)	↑(+)* (2yrs) ⊗ (3yrs)					Home intervention was effective in extending survivorship up to 3.5 years. However, by 3 years, mortality rates between the intervention and control groups were no longer significantly different.
9.	Ploeg et al. (2010)	Primary 1. QALYs (The Health Utilities Index Mark 3) Hearing Speech Ambulation Dexterity Emotion Cognition Pain/discomfort Secondary 2. Self-rated health (SF-36) 3. Functional status (Older Americans Resources and Services Multidimensional Functional Assessment Questionnaire) ADL IADL 4. *Cost of healthcare utilisation*, *including prescription drugs and nursing cost (Health and social service utilisation survey)*	⊗ ⊗ ⊗ ⊗					The mean difference in QALYs, self-rated health, overall cost of healthcare cost and functional status and self-rated health between IG and CG were not statistically significant. PHV have not shown positive results for the studied population either for general population of segregated by low and high risk of functional decline.
10.	van Hout et al. (2010)	Primary 1. IADL disability (Groningen Activity Restriction Scale) 2. Functional health (COOP- WONCA charts (12) and short Form 36 item (SF-36) Secondary 3. Hospital admission/Institutionalisation (extracted from the local hospital registry, supplemented with self-report data) 4. Nursing home/disabled home admission (homes’ registries or in PCPs’ medical records) 5. Mortality (checked with the PCPs and the hospital database)	⊗ ⊗ ⊗ ⊗ ⊗					There is no significant difference among the IG and CG for the outcome measures except for the higher risks of hospitalisation among the IG in the subgroup with poor health status. In general, the study did not prevail effect of home visits by the nurses among the frail population. However, hospital admission risks among the participants with poorer health should be further investigated with more holistic approaches.
11.	Behm et al. (2014)	Primary 1. Morbidity (Cumulative Illness Rating Scale for Geriatrics (CIRS-G)) Secondary 2. Symptoms (Göteborg QoL Instrument (GQL)) 3. SRH (Item 1—SF-36)	↓(+)* ⊗ ⊕^c^	Secondary Less satisfaction with health–physical and psychological (Fugl-Meyer LiSat-11)	↓(+)*			It is possible to postpone decline in health outcomes measured as morbidity, SRH and satisfaction with health among participants at risk of frailty by providing multidimensional and multi-professional approach in PHV and SM intervention. SM had greater effect on SRH for one year than in 2 years follow-up.
12.	Godwin et al. (2016)	Primary 1. Symptomology (Comorbidity Symptom Scale) Secondary 2. Healthcare utilisation Visit to ED Visit to family physician Utilisation of diagnostic services	⊗ ⊗	Primary 1. Satisfaction with care (PSQ-18) Secondary 2. HRQoL (SF-36, CASP-19) emotional health emotional well-being 3. Anxiety/depression—included in symptomology assessment (Comorbidity Symptom Scale)	⊕^d^ ⊗ ⊗	Secondary 1. HRQoL (SF-36) Social functioning	⊗	The intensive 1-year care management programme (ElderCare project) was not have an impact on QoL, symptomology and satisfaction with care among the studied participants.
13.	Kono et al. (2016)	1. Physical functioning (ADL)	↑(+)*	Care need level Daily life satisfaction	↑(-)* ↓(-)	1. Long term care service utilisation (the LCTI system)	⊗	There is significant difference of ADL functioning and care need level among the IG as compared to CG over 24 months. PHV could be effective in prevention of ADL and care needs dependency and the effects could be seen up to 1 year after the programme ends.
14.	Sherman et al. (2016)	General outcome SRH (78-questions self-reported questionnaire at baseline and follow ups) Health index Energy, mood, fatigue, vertigo, bowel function Pain Mobility Perceived health General health Health problems Use of medication Knowledge about healthcare services (3 questions on knowledge of home-help service and leisure time activities and another 3 questions on knowledge about their contact with local HCC and DN and two about home-help service, both current and past)	↓(-)* ↓ (+)* ↓(-)* ⊗ ⊗ ↑(-)* ↑(+)*	General outcome SRH (78-questions questionnaire) Health Index Well-being Loneliness Health behaviour Physical activity Smoking Eating habit Alcohol intake BMI Experience and usefulness of PHV (additional 2 questions during PHV follow-ups)	⊗ ↓(-)* ↓(-)* ⊗ ⊗ ↑(-)* ⊗ ↑(+)			Participants from both IG and CG experienced decreased health and well-being. Among the IG, PHV was effective in reducing pain, increasing knowledge, visits to healthcare centres and medication intake. However, there is no significant effect on health behaviour. 84% of the participants agreed on the usefulness of PHV.
15.	Arola et al. (2018)			1. Total score of sense of coherence (SoC) (3-item Orientation to Life Questionnaire (SOC-13): Comprehensibility Manageability Meaningfulness	↑(+)*^e^			PHV intervention helps migrants who have lived in host countries for a long-time cope with ageing-related challenges better. They have a SoC similar to the native-born older adults.
16.	van Lieshout et al. (2018)	Primary Functional ability ADL (Katz-6) Secondary Functional ability IADL (GARS) Functional capacity (Six-meter walk test (6MWT)) Physical fitness Muscle strength (Handgrip strength) Walking speed (TUG) Medication (medication review using the Prescribing Optimization Method (POM)) Mobility (Morton Mobility Index) Nutritional status (SNAQ) Healthcare utilisation (Question on hospital admission and services used) hospital admission nursing home admission primary care visits receiving home care	⊗ ↑(+)* ↑(+)* ⊕^f^ ↑(+)* ↑(-) ⊗ ⊗ ⊗	Secondary QoL (SF-12) physical and mental status Depression (HADS) Loneliness (Jong Gierveld Loneliness Scale)	⊗ ⊗ ⊗			The proactive multicomponent intervention programme (SPRY) was not effective in reducing daily functioning, improving QoL or increasing healthcare consumption among the pre-frail older adults at the one-year follow-up. However, IG participants experienced improvements in walking speed, functional capacity and IADL.
17.	Limatta et al. (2019)	Primary Mortality (data from central registry)	⊗	Secondary HRQoL (15-dimensional assessment scale (15D))	⊕^g^			PHV slowed down the decline in HRQoL among the IG in the first year, but the effect diminished at the end of the second year.
18.	Szanton et al. (2019)	Primary Functional disability ADL at 5 months IADL at 5 months Secondary Functional disability ADL at 12 months° IADL at 12 months	↓(+)* ⊗ ⊗ ⊗	Secondary 1. Perceived benefit of PHV	↑(+)			CAPABLE programme has effectively decreased disability among the IG. Disability may be modifiable by addressing both individual and environmental factors.
19.	Hoedemakers et al. (2022)^h^	General Physical function (Katz-15) Burden of medicine (Living with Medicines Questionnaire) Continuity of care (Nijmegen Continuity Questionnaire, team and cross boundary continuity domain43 + HoClient Perceptions of Coordination Questionnaire) *Cost* *Health*, *social and informal care (institute for Medical Technology Assessment (iMTA) Medical Consumption Questionnaire)* *Medication (Prescriptions in patient records extracted from GP information system)* *Bundle payment of chronic care programme (• Care chain information system ‘Care2U’)*	↓(-) ↑ (-) ↑ (-) ↑(-)	General Psychological well-being (Mental Health Inventory) Enjoyment of life (Investigating Choice Experiments for the Preferences of Older People) Resilience (Brief Resilience Scale (BRS)) Person-centredness (Person- centred Coordinated Care Experience Questionnaire, experience of person-centred care domain) Autonomy (Pearlin Mastery Scale)	↓(-) ↑ (+) ↓(-) ↑ (+) ↓(-)	General 1. Social relationship and participation (Impact on Participation & Autonomy, social life and relationships domain)	↓(-)	Based on MCDA analysis, the CCFE intervention is the preferred programme as evaluated by four stakeholders as compared to usual care at 6 months, driven by person-centredness and enjoyment of life. However, at 12 months, the score for CCFE preference was lower from payer’s and policy maker’s perspective driven by deterioration of physical health and higher cost for programme execution.
20.	Blotenberg et al. (2023)^i^	General HRQoL (SF-12) Physical health (general) Physical health (affected by COVID-19)	↑(+) only after 1^st^ PHV and decrease afterwards ⊗	General HRQoL Mental health (general) Mental health (affected by COVID-19)	↑(+) steadily from 1^st^ to 3^rd^ PHV but slightly decrease after 4^th^ PHV ↓(-)			PHV has been a successful nursing intervention among the population of the older adults in this study and should be further researched in term of the definition and concept. There was significant difference of the mental health mean variance among the participants affected by COVID-19 as compared to those not affected. Based on this study’s outcomes, PHV intervention is recommended as part of regular healthcare services in Germany. Specific criteria should be considered to address specific exception effects that influenced the outcome measures.

Indicator for the findings: ↑ = increase; ↓ = decrease; ⊗ = no change/difference; (+) = positive change/improvement; (-) = negative change/worsen; * = statistical significance (p<0.05);

^a^: The effect on health behaviour were limited to only two out of 11 assessed items which the participants in the intervention group ceased smoking and cared for their oral health than in the control group.

^b^: No significance different in total cost. However, cost per person was significantly lower prescription medications among the intervention group participants

^c^: The odds of deteriorating in SRH were significant lower for the participants in the senior meeting group as compared to control group in the first year. No significance difference between the groups in second year.

^d^: There were no significant difference in general patients’ satisfaction with health between both groups except on accessibility and convenience

^e^: There was a significant difference in total SOC score favouring IG in 6 months but no significant different between the groups in 12 months.

^f^: There was significant difference in walking speed and functional capacity among IG at 6 months. Right HGS was improved at 6-months follow-up although not significant while left HGS worsen at 6-months follow-up and reduced significantly after 6-months follow-up.

^g^: There was significantly different in HRQoL increment among the IG group in the 1^st^ year but the effect diminished in 2 years.

^h^: Outcomes measured were based on standardised performance score of the intervention programme by using multi-criteria decision analysis.

^i^: Comparison of the outcomes for the longitudinal data and mean variance (MV) at each time of data collection point between the studied population and general German older adults’ population norm sample 1994. An exceptional effect of COVID-19 on HRQOL were analysed.

Abbreviation:

IG: Intervention group; CG: Control group

GDS—15: Geriatric Depression Scale, 15 items

MMSE: Mini Mental State Examination

SMAF: Functional Autonomy Measurement System

SNAQ: Short Nutritional Assessment Questionnaire

GARS: Groningen Activity Restriction Scale

TUG: Timed Up and Go

HADS: Hospital Anxious Depression Scale

CAPABLE: Community Aging in Place—Advancing Better Living for Elders

#### Resilience as PHV outcome measure

Among all studies, a quasi-experimental study conducted by [79] is the only one that assessed resilience as one of the outcomes. Interestingly, this study evaluated the effectiveness of PHV from several viewpoints. The authors investigated if the Care Chain Frail Elderly (CCFE), a comprehensive and person-centred integrated care plan, was preferred by patients, informal caregivers, professionals, payers and policymakers. The study revealed that CCFE programme was found to be more favourable as a six-month intervention compared to a 12-month intervention. This is because the longer duration led to a decline in physical functioning and increased expenses. However, measurements of resilience revealed no significant difference was found between the intervention and control groups. In addition to resilience, this study also assessed autonomy. However, no significant difference was found between the groups.

### Health-related outcomes

#### Functional ability

Functional ability or impairment were the most frequently measured outcomes and appeared in 11 studies [61–63, 65, 67, 69, 70, 73, 76, 78, 79]. [79] examined physical functioning of the participants while [67] evaluated the overall functional health. [62] reported positive findings regarding the likelihood of being independent in performing ADLs at three years, which was significantly low for the intervention group (adjusted odd ratio (OR) 0.4; 95% confidence interval (CI), 0.2 to 0.8; p = 0.02). Similarly, [76] found significant improvement in IADL after one year follow-up (Friedman’s test p<0.04, X2 = 33.29). The research team also observed a 30% reduction in ADL disability after 5 months (relative risk (RR), 0.70; 95% CI, 0.54–0.93; p = 0.01). Nonetheless, the ADL assessment in the same study produced insignificant results thoughout12-month follow-up.

#### Mortality

Mortality was examined in five studies [61, 63, 65, 68, 70] and has been chosen as an indicator of survivorship for the ABLE programme participants (Advancing Better Living for the Elderly) in the study conducted by [68]. Survivorship was the only outcome measured this study and represented by the mortality rate. The mortality rate in the intervention group was significantly low for a period up to 3.5 years. After the four-year follow-up, there was no significant difference between the intervention and the control group.

#### Risk of institutionalisation and continuity of care services

The outcomes related to institutionalisation were categorised as risk of hospitalisation or institutionalisation [61, 62, 70], visit to a physician or primary care provider [62, 63, 72, 76], nursing home admission [62, 63], accessibility to healthcare services [61, 65, 74, 76], maintaining continuity of care [79] or utilising of home care services [76]. In the study by [62], the team found that the risk of permanent nursing home admission was significantly lower in the intervention group compared to the control group (p = 0.02). In another study with a different population and context, they found the in-home visit significantly increased visits to primary care institutions among the intervention group (p = 0.05) [63]. In the study conducted by [79], the intervention group had significantly higher level of continuity of care compared to the control group. No notable disparities were found among participants in the remaining results.

#### Other health-related outcomes

The research included in this review exhibited heterogeneity in outcomes in addition to the primary health-related outcomes. The investigations encompassed factors such as mobility [66, 76], morbidity [71], medication intake and nutritional status [76], influenza vaccination [62], mental health function [65, 67], symptoms [71, 72] and physical fitness [76]. After two years of follow-ups, both PHV and SM groups were found to be effective in delaying morbidity in both intervention groups with odd ratio 0.60 (p = 0.035, 95% CI = 0.37–0.96) for the PHV and 0.52 (p = 0.008, 95% CI = 0.32–0.84) for the SM group [71]. However, in the same study, no significant difference was found in the progression of symptoms between the groups. Symptomology outcomes also did not differ significantly between the intervention and control groups [60]. In the study by [76], polypharmacy among the intervention group was reported at 63.1% (used five or more medicines) and 26.9% (either two, three or four medicines). Within the same study, there were no significant differences observed in terms of physical fitness and nutritional status across the groups. The intervention group had a higher influenza vaccination rate compared to the control group (33% vs 25%, p = 0.01). this effect is only observed in a particular intervention group that has low risk of nursing home admission [63].

The subgroup analysis conducted in three specific region hat received treatments from different interveners (Zone A, B and C) revealed that the vaccination rate among the individuals in the intervention group participants in Zone A and B has increased (39% vs 24%, p = 0.01) but not in Zone C (26% vs 29%, p = 0.37) [63]. This research demonstrated that the effect of health literacy and health behaviour differs based on the proficiency of the interveners and their personalised expertise in providing care and delivering health education during the intervention. In terms of mental health, [67] discovered significant improvement in the mental health component based on the SF-36 Quality of Life assessment. In contrast, [65] reported unfavourable outcomes in their study on cognitive function.

### Psychology-related outcomes

The most commonly assessed psychological dimensions were depression, anxiety, loneliness and QoL [61, 64–67, 72, 76, 77]. In addition, the researchers assessed various aspects of an individual’s overall state of health including general well-being [61, 65], psychological well-being [79], health behaviour [66], coping ability [67], SoC [75], satisfaction with health or life [71, 73], satisfaction with care [72], experience and usefulness of PHV [74] as well as enjoyment of life, person centeredness, resilience and autonomy [79]. Interestingly, the most recent study by Blotenberg and colleagues [80] examined mental health using HRQoL questionnaire for two distinct groups: general group and a group impacted by COVID-19. Participants affected by COVID-19 have a lower mean variance in mental health compared to those who are not affected.

The study found positive results in several areas. Firstly, there was a significant reduction in depression (p = 0.009), Secondly, both intervention groups the research conducted by Behm et al. [71] showed lower likelihood to becoming less satisfied with their health (OR 0.43, p = 0.013, 95% CI = 0.22–0.84 for the PHV and OR 0.28,p = 0.001, 95% CI = 0.14–0.59 for the SM group). Thirdly, 60% of the participants reported that the PHV was either very useful or useful [74]. Fourthly, according to [78], 91.6% of the participants stated that the intervention had benefited them. Lastly, there were positive scores of enjoyments of life (standardised performance score 0.729) and person-centredness (standardised performance score 0.749) [79]. At six months follow-up, a study assessing SoC found a significant difference in the total SoC score favouring the intervention group (p = 0.038) among the specific migrant community in Sweden [75].

While the majority of the measured outcomes, such as QoL, health behaviour, well-being, satisfaction with the programme, satisfaction with care, loneliness, depression or anxiety, and coping did not show a significant difference among two or more distinct groups, a few outcomes did show a negative effect, especially psychological health, resilience and autonomy. In this study, the control group had a higher standardised performance score for resilience compared to the intervention group [79].

### Social-related outcomes

Seven studies examined social-related outcomes. The outcome measures included the utilisation of community care or long-term care [61, 62, 73], as well as the assessment of social relationships, functioning and social participation [64, 79] which encompassed factors such as social support and social integration [65, 67] and engagement rate [67]. Additionally, one study evaluated social functioning as part of the assessment of QoL [72].

There was a significant difference in enhanced perceived social support among frail older adults who participated in proactive nursing health promotion interventions (P = 0.009) [67]. Although there was no statistically significant difference, the studies found positive results in terms of social relationships and social participation [79] and community care utilisation [61].

### Financial implication

In addition to the assessment of the impact of PHV on health, psychological and social outcomes, this review also analysed the financial implications as a result of an effective PHV intervention that involved five studies. The primary cost evaluation focused on the utilisation of healthcare and social care, as measured in the study by [69]. Similarly, [79] evaluated the cost associated with informal care, as well as the fundamental costs of basic health and social care. They also calculated the medication and the bundle payment of the chronic care programme as part of miscellaneous expenses. In another study, [62] calculated fundamental cost based on total savings resulting from programme implementation. In their earlier study, the team transformed the cost of the intervention into disability-free life years and the avoidance of one day of hospital admission. They discovered that the cost of an intervention programme to offset one year of disability-free life was $6000, while the cost to avoid one day of hospitalisation was $38. While the total cost did not differ significantly between the two groups, the intervention group exhibited lower per-person expenditures compared to the control group. In the following study, the team revealed that the PHV intervention resulted in a favourable return on investment, as indicated by the overall cost savings [63]. Financial repercussions were observed in healthcare, social care and informal care [61, 79], as well as in medicine and expenditure on the chronic care bundle payment plan [79].

### Methodological concerns

This review covered studies of both high and medium quality. It is observed that inconsistent results were due to the heterogeneity of study participants, PHV components, the involvement of multidisciplinary team members with varied levels of experience and expertise, assessment tools, and intervention duration. The heterogeneity led to a high degree of uncertainty regarding clinical questions and had an ambiguous clinical impact. The majority of the studies included in this review involved older adults who are in good health and came from developed countries in the Western continents and only two studies represented Asia region (Japan). Therefore, the studies’ generalisability is deemed satisfactory and future research anticipating the Eastern context is expected to represent the uniqueness of sociodemographic characteristics as well as physical, psychological and social (psychosocial) health of its older population.

## Discussion

Since the end of the 20th century, a plethora of studies on PHV interventions, systematic reviews [15, 17, 19, 20, 23, 81, 82] and umbrella review [18] have been published. While most prior systematic reviews have focused on health-related outcomes, contemporary research has broadened its scope to include the psychosocial domain [22, 81].

Randomised controlled trials (RCTs) are the predominant research methodology. The existing body of knowledge continues to rely heavily on health-related outcomes. This review is evident that the psychosocial domain is still lacking sufficient research to contribute to health promotion and disease prevention for the older adult population [77, 83], In addition, only minimal attention is given to the assessment of the cost-effectiveness and economic impact associated with the implementation of PHV.

Overall, PHV interventions have demonstrated positive outcomes in healthy populations but have not been effective in improving health of the frail older adults. This finding is consistent with earlier systematic reviews that emphasised the need for developing targeted care plans and identifying the optimal intervention periods for high-risks populations [17, 84, 85].

In terms of PHV effectiveness, our findings are consistent with previous research that highlights inconsistent or ambiguous evidence of the effectiveness. The inconsistency can be the result of a diverse study design such as the protocol, duration, numbers and skills of the interveners, study participants and outcome measures [15, 19, 20, 22, 82, 86].

### PHV designs and approaches

The literature on PHV intervention recognises the involvement of a team of multidisciplinary professionals in carrying out home visits. The visits involve comprehensive health assessment, aiming for specific outcomes in improving the health and well-being of the participants. There have been issues raised about the variation in PHV designs and approaches [63, 74], especially the comparison between structural and flexible approaches [65]. Specific approaches can be seen in [66], the nurses utilised the Minimum Data Set-Home Care 2.0 (MDS-HC) as an alternative to the CGA which was not feasible in the Japanese context. A structured protocol can also be observed based on the assessment of 12 health domains and referring patients to general practitioners (GPs) or other healthcare professionals for specific concerns, such as rehabilitation programme [65]. Proactive nursing health promotion is based on the model of vulnerability as the conceptual approach [67]. Several large-scale intervention programmes with well-established protocols and a few with preliminary studies’ results serve as references for improving the current research on PHV effectiveness [68, 72, 75, 76, 78, 79]. In addition to home visits, group meetings were implemented as an intervention to promote social interaction and facilitate in-depth discussion and understanding of person’s health issues [71, 74–76]. Involving older adults in the design of an intervention programme that aimed to enhance their resilience will contribute to the improvement of the programme [87].

Despite there were variety of designs and approaches used for PHV, none of the studies focused on evaluating PHV assessment or thoroughly considering the necessity of redesigning the intervention for better effectiveness and efficiency. The only exception was a specific discussion and evaluation on how the study’s outcomes varied based on the experience of the interveners, highlighting the importance of specific training to enhance the their performance [63]. In addition, [19, 88] emphasised the significance of incorporating process evaluation into PHV design to avoid intervention infidelity.

In relation to the duration of PHV, 11 out of the 20 interventions included in this review had an implementation duration ranging from five to 12 months. Out of these studies, only three have shown positive results [62, 71, 80]. Based on the findings of this review, we were unable to determine whether a longer intervention duration is better or the opposite. Undoubtedly, additional parameters such as the frequency of follow-up sessions and the length of each individual session hold equal significance.

### Outcome measures

Each study in this review addressed at least four primary outcomes and more than five additional secondary outcomes related to health and psychology domains. Social domain on the other hand only involved a single measurement in each of seven studies which included either social support, social functioning, social relationship and participation, community care or long-term care utilisation [61, 63–65, 72, 73, 79]. However, [67] examined two outcomes namely engagement rate and perceived social support. Limited information on the social domains has shown that there is a scarcity of research on PHV focusing on psychosocial outcomes, especially when compared to research on the effectiveness of PHV on health-related outcomes.

Instead of commonly used validated assessment tools, such as the SF-36, MMSE, CASP-19, GDS, Katz and Lawton Brody, and PSQ, alternative assessment tools such as Fugl-Meyer LiSat-11 and the Goteborg Quality of Life Instrument were used in [71], and Dupuy’s General Well-being Schedule (GWBS) in [65]. The adoption of these instruments without providing detailed justification or referencing their validity and reliability could contribute to methodological bias. Moreover, the subjective evaluation of self-rated health is strongly influenced by how the participants perceive their own health. As a result of ageism, older participants may assess their health in accordance with their advancing age, with the prevailing belief that “being old means being sick” [89]. The findings of this review show that none of the three studies found a significant difference in self-perception of health and age preference between the intervention and control groups [61, 71, 74].

### The effectiveness of PHV on resilience

Older adults possess protective characteristics that have been developed during a lifetime after surviving a series of adversities throughout their life course, making them more resilient than younger individuals [24, 90]. The term used to describe the benefits gained is emotional maturity which assists older adults managing challenges or coping effectively [76]. Interestingly, another factor identified to improve coping skills is reduced exposure to unpleasant news through excessive use of social media [28]. However, there are conflicting findings indicating that the younger older adults exhibit greater resilience as a result of declining health or the abandonment of favourite pastimes, which may divert their attention from excessive rumination on the negative events they are presently confronting [77]. Due to the absence of the RCTs and only one quasi-experimental study on resilience [79], we were unable to make any definitive conclusions on the effectiveness of PHV on resilience for the older adults population.

Academic scholars and public policymakers are emphasising the importance of resilience research as part of individual and societal efforts to overcome adversity [91–94]. An older adult who is resilient may experience improvement in their physical and mental health, increased physical activity and a better ability to tolerate pain or discomfort, as well as psychological benefits such as an enhanced QoL [95, 96]. It is necessary to conduct multidimensional research on resilience that focuses on health and non-health attributes to identify specific stressors and positive outcomes that can help enhance resilience. Additionally, it is important to assess the impact of societal factors on personal resilience. The research should take into consideration the P4 medicine strategy, which is preventive, predictive, personalised, and participatory elements in health interventions [95].

### Strengths and limitation

This systematic review has several advantages in comparison to reviews. Firstly, we provide an in-depth multidomain analysis to assess the effectiveness of PHV. We analysed the findings of prior research from a broader perspective and emphasised the importance of a holistic approach in designing multidimensional PHV interventions that encompass biopsychosocial health approach rather than focusing solely on preventing specific diseases. Secondly, we meticulously conducted the literature search by including grey literature search on top of a comprehensive online database search, which enhanced the comprehensiveness of the analysis. Methodologically, we scrutinised the research evidence using the NHMRC framework to rigorously evaluate the body of evidence in each study.

Despite of several advantages and uniqueness of this systematic review as compared earlier reviews related to PHV implementation, it is still constrained in certain aspects. Although this systematic review was designed according to the procedure outlined by PRISMA 2020 guideline, we are unable to completely exclude certain biases. No restrictions on language, time period and inclusion of diverse set of synonyms for the search keywords following the PICO framework, has produced an extensive volume of results. Substantial amount of data led to longer screening duration, making it inevitable that several important record could not be missed. Furthermore, the selection of keywords for resilience in this review may have overlooked other relevant concepts such as hardiness, grit, community support or contextual dynamics like culture and norms that may influence health behaviour. Due to the diversity in the intervention design, outcomes and measurement tools, it was not feasible to directly compare the effectiveness of PHV on health, psychological and social outcomes, specifically resilience. Due to inconclusive findings for this review and the paucity of specific research on the effectiveness of PHV on resilience, we were unable to establish a definite and robust measure of the effectiveness of PHV on resilience among the older adults population.

## Conclusion

In this review, we found that PHV intervention placed a significant emphasis on health-related outcomes, which aligned with previous research on this subject. One noteworthy aspect of this study is that it measured the effectiveness of PHV on resilience, which is relatively unexplored area of research. This warrants continuous research on the structured multidimensional PHV that can boost, improve or maintain resilience among older adults. Furthermore, contextual dynamics, within and beyond family support and extension to the community level would enhance the analysis of resilience. In order to comprehensively examine resilience, researchers must also consider both universal (etic) and cultural-specific (emic) factors that influence human behaviour in relation to maintaining and caring for their health [97–99]. Providing a person-centred PHV design requires structural changes and public policy revision to expand training and provide more opportunities for professional qualifications to perform PHV efficiently and effectively. From a methodology standpoint, incorporating technological advancements and artificial intelligence could be considered in the design of a PHV and the person-centric healthcare plan can boost its feasibility, practicality and integration across various healthcare services and providers while also making it more technologically friendly. Lastly, financial assessments such as costs and benefits analysis can provide more objective evaluation of PHV effectiveness, which will benefit the targeted population, society and the government as public healthcare providers.

## Supporting information

S1 File PRISMA 2020 checklist. (DOCX)

S2 File Keyword stings syntax. (DOCX)
