# Supplementary material for: The effectiveness of preventive home visits on resilience and health-related outcomes among community dwelling older adults: A systematic review
Source: PLoS One. 2024 Jul 1;19(7):e0306188. doi: 10.1371/journal.pone.0306188 (PMC11216600; doi:10.1371/journal.pone.0306188)
Supplement: S2 File — (DOCX) [file pone.0306188.s002.docx]

**S2 Supplementary Material: Keyword strings syntax**

PICO framework and the keywords string

| **PICO elements** | | **Keywords and synom** |
| --- | --- | --- |
| Participants (P) | **Community dwelling older adults aged 60 years and above** who receive PHV or any related programme. Pre-frail and frail older adults were included in the review. | older adult* OR older people OR older person* OR aged OR elder* OR elder* people OR elder* person* OR senior citizen OR old* citizen |
| Intervention (I) | Studies were included if the intervention involved **preventive home visits** or any other type of home care provided by trained nurses or a multidisciplinary team consisting of a nurse, physical therapist, occupational therapist, social worker or other professionals such as a physician, dietitian, pharmacist or psychologist. In addition to regular home visits, intervention may include group meetings between participants and healthcare providers in order to increase health literacy and promote social participation. | preventive home visit* OR home visit* OR home care OR homecare OR house call |
| Comparator (C) | Those who received **usual care** or **no intervention** throughout the programme. However, the absence of a comparator for non-RCT studies is acceptable. | - |
| Outcomes (O) | 1. Resilience 2. Health related outcomes including quality of life and unmet needs | resilien* OR coping OR adversit* OR bounce back OR positive adaptation OR protective factor*  AND  health status OR health outcome* OR disabilit* OR impair* OR frail* OR health related quality of life OR HRQoL OR quality of life OR unmet need* OR met need* OR need* |

Keyword search for each database

| **Database** | **Keywords** | **Results** |
| --- | --- | --- |
| WOS | (older adult* OR older people OR older person* OR aged OR elder* OR elder* people OR elder* person* OR senior citizen OR old* citizen**)** (All Fields) and (preventive home visit* OR home visit* OR home care OR homecare OR house call) (All Fields) and (resilien* OR coping OR adversit* OR bounce back OR positive adaptation OR protective factor*) (All Fields) and (health status OR health outcome* OR disabilit* OR impair* OR frail* OR health related quality of life OR HRQoL OR quality of life OR unmet need* OR met need* OR need*) (All Fields) | 2421 |
| Scopus | ALL ( ( older AND adult* OR older AND people OR older AND person* OR aged OR elder* OR elder* AND people OR elder* AND person* OR senior AND citizen OR old* AND citizen ) AND ( resilien* OR coping OR adversit* OR autonomy OR bounce AND back OR positive AND adaptation* OR protective AND factor* ) AND ( preventive AND home AND visit* OR preventive AND home AND care OR home AND visit* ORhome AND care OR homecare OR home AND health AND care OR home AND healthcare OR house AND call OR home AND nursing ) AND (health AND status OR health AND indicator* OR health AND outcome* OR disabilit* OR impair* OR frail* OR health AND related AND quality AND of AND life OR hrqol OR quality AND of AND life OR qol OR unmet AND need* OR met AND need* OR need* ) ) | 261 |
| PubMed | (((("older"[All Fields] OR "olders"[All Fields]) AND "adult*"[All Fields]) OR (("older"[All Fields] OR "olders"[All Fields]) AND ("people s"[All Fields] OR "peopled"[All Fields] OR "peopling"[All Fields] OR "persons"[MeSH Terms] OR "persons"[All Fields] OR "people"[All Fields] OR "peoples"[All Fields])) OR (("older"[All Fields] OR "olders"[All Fields]) AND "person*"[All Fields]) OR ("aged"[MeSH Terms] OR "aged"[All Fields]) OR "elder*"[All Fields] OR ("elder*"[All Fields] AND ("people s"[All Fields] OR "peopled"[All Fields] OR "peopling"[All Fields] OR "persons"[MeSH Terms] OR "persons"[All Fields] OR "people"[All Fields] OR "peoples"[All Fields])) OR ("elder*"[All Fields] AND "person*"[All Fields]) OR ("aged"[MeSH Terms] OR "aged"[All Fields] OR ("senior"[All Fields] AND "citizen"[All Fields]) OR "senior citizen"[All Fields]) OR ("old"[All Fields] AND ("citizen"[All Fields] OR "citizen s"[All Fields] OR "citizens"[All Fields]))) AND ((("prevent"[All Fields] OR "preventability"[All Fields] OR "preventable"[All Fields] OR "preventative"[All Fields] OR "preventatively"[All Fields] OR "preventatives"[All Fields] OR "prevented"[All Fields] OR "preventing"[All Fields] OR "prevention and control"[MeSH Subheading] OR ("prevention"[All Fields] AND "control"[All Fields]) OR "prevention and control"[All Fields] OR "prevention"[All Fields] OR "prevention s"[All Fields] OR "preventions"[All Fields] OR "preventive"[All Fields] OR "preventively"[All Fields] OR "preventives"[All Fields] OR "prevents"[All Fields]) AND ("home environment"[MeSH Terms] OR ("home"[All Fields] AND "environment"[All Fields]) OR "home environment"[All Fields] OR "home"[All Fields]) AND "visit*"[All Fields]) OR (("home environment"[MeSH Terms] OR ("home"[All Fields] AND "environment"[All Fields]) OR "home environment"[All Fields] OR "home"[All Fields]) AND "visit*"[All Fields]) OR ("nursing"[MeSH Subheading] OR "nursing"[All Fields] OR ("home"[All Fields] AND "care"[All Fields]) OR "home care"[All Fields] OR "home care services"[MeSH Terms] OR ("home"[All Fields] AND "care"[All Fields] AND "services"[All Fields]) OR "home care services"[All Fields] OR ("home"[All Fields] AND "care"[All Fields])) OR "homecare"[All Fields] OR ("house calls"[MeSH Terms] OR ("house"[All Fields] AND "calls"[All Fields]) OR "house calls"[All Fields] OR ("house"[All Fields] AND "call"[All Fields]) OR "house call"[All Fields])) AND ("resilien*"[All Fields] OR ("adaptation, psychological"[MeSH Terms] OR ("adaptation"[All Fields] AND "psychological"[All Fields]) OR "psychological adaptation"[All Fields] OR "coping"[All Fields] OR "coped"[All Fields] OR "copes"[All Fields] OR "copings"[All Fields]) OR "adversit*"[All Fields] OR (("bounce"[All Fields] OR "bounced"[All Fields] OR "bounces"[All Fields] OR "bouncing"[All Fields]) AND ("back"[MeSH Terms] OR "back"[All Fields])) OR (("positive"[All Fields] OR "positively"[All Fields] OR "positiveness"[All Fields] OR "positives"[All Fields] OR "positivities"[All Fields] OR "positivity"[All Fields]) AND ("acclimatization"[MeSH Terms] OR "acclimatization"[All Fields] OR "adaptation"[All Fields] OR "adaptations"[All Fields] OR "adapt"[All Fields] OR "adaptabilities"[All Fields] OR "adaptability"[All Fields] OR "adaptable"[All Fields] OR "adaptational"[All Fields] OR "adaptative"[All Fields] OR "adapte"[All Fields] OR "adapted"[All Fields] OR "adapting"[All Fields] OR "adaption"[All Fields] OR "adaptions"[All Fields] OR "adaptive"[All Fields] OR "adaptively"[All Fields] OR "adaptiveness"[All Fields] OR "adaptivity"[All Fields] OR "adapts"[All Fields])) OR (("protect"[All Fields] OR "protected"[All Fields] OR "protecting"[All Fields] OR "protection"[All Fields] OR "protections"[All Fields] OR "protective agents"[Pharmacological Action] OR "protective agents"[MeSH Terms] OR ("protective"[All Fields] AND "agents"[All Fields]) OR "protective agents"[All Fields] OR "protectant"[All Fields] OR "protectants"[All Fields] OR "protective"[All Fields] OR "protectively"[All Fields] OR "protectiveness"[All Fields] OR "protectives"[All Fields] OR "protects"[All Fields]) AND "factor*"[All Fields])) AND ("health status"[MeSH Terms] OR ("health"[All Fields] AND "status"[All Fields]) OR "health status"[All Fields] OR (("health"[MeSH Terms] OR "health"[All Fields] OR "health s"[All Fields] OR "healthful"[All Fields] OR "healthfulness"[All Fields] OR "healths"[All Fields]) AND "outcome*"[All Fields]) OR "disabilit*"[All Fields] OR "impair*"[All Fields] OR "frail*"[All Fields] OR ("quality of life"[MeSH Terms] OR ("quality"[All Fields] AND "life"[All Fields]) OR "quality of life"[All Fields] OR ("health"[All Fields] AND "related"[All Fields] AND "quality"[All Fields] AND "life"[All Fields]) OR "health related quality of life"[All Fields]) OR ("hrqols"[All Fields] OR "quality of life"[MeSH Terms] OR ("quality"[All Fields] AND "life"[All Fields]) OR "quality of life"[All Fields] OR "hrqol"[All Fields]) OR ("quality of life"[MeSH Terms] OR ("quality"[All Fields] AND "life"[All Fields]) OR "quality of life"[All Fields]) OR ("unmet"[All Fields] AND "need*"[All Fields]) OR ("met"[All Fields] AND "need*"[All Fields]) OR "need*"[All Fields])) AND ((clinicaltrial[Filter] OR meta-analysis[Filter] OR randomizedcontrolledtrial[Filter]) AND (humans[Filter])) | 1025 |
| MEDLINE | 1=(older adult* OR older people OR older person* OR aged OR elder* OR elder* people OR elder* person* OR senior citizen OR old* citizen).mp. [mp=title, abstract, heading word, drug trade name, original title, name of substance word, subject heading word, floating sub-heading word, keyword heading word, organism supplementary concept word, protocol supplementary concept word, rare disease supplementary concept word, unique identifier, synonyms]  2=(resilien* OR coping OR adversit* OR bounce back OR positive adaptation OR protective factor).mp. [mp=title, abstract, heading word, drug trade name, original title, name of substance word, subject heading word, floating sub-heading word, keyword heading word, organism supplementary concept word, protocol supplementary concept word, rare disease supplementary concept word, unique identifier, synonyms]  3=(preventive home visit* OR home visit* OR home care OR homecare OR house call).mp. [mp=title, abstract, heading word, drug trade name, original title, name of substance word, subject heading word, floating sub-heading word, keyword heading word, organism supplementary concept word, protocol supplementary concept word, rare disease supplementary concept word, unique identifier, synonyms]  4=(health status OR health outcome* OR disabilit* OR impair* OR frail* OR health related quality of life OR HRQoL OR quality of life OR unmet need* OR met need* OR need*).mp. [mp=title, abstract, heading word, drug trade name, original title, name of substance word, subject heading word, floating sub-heading word, keyword heading word, organism supplementary concept word, protocol supplementary concept word, rare disease supplementary concept word, unique identifier, synonyms]  5= 1 and 2 and 3 and 4 | 317 |
| CINAHL | (older adult* OR older people OR older person* OR aged OR elder* OR elder* people OR elder* person* OR senior citizen OR old* citizen) AND (resilien* OR coping OR adversit* OR bounce back OR positive adaptation OR protective factor*) AND (preventive home visit* OR home visit* OR home care OR homecare OR house call) AND (health status OR health outcome* OR disabilit* OR impair* OR frail* OR health related quality of life OR HRQoL OR quality of life OR unmet need* OR met need* OR need*) | 441 |
| Embase | 1=(older adult* OR older people OR older person* OR aged OR elder* OR elder* people OR elder* person* OR senior citizen OR old* citizen).mp. [mp=title, abstract, heading word, drug trade name, original title, device manufacturer, drug manufacturer, device trade name, keyword heading word, floating subheading word, candidate team word]  2=(resilien* OR coping OR adversit* OR bounce back OR positive adaptation OR protective factor).mp. [mp=title, abstract, heading word, drug trade name, original title, device manufacturer, drug manufacturer, device trade name, keyword heading word, floating subheading word, candidate team word]  3=(preventive home visit* OR home visit* OR home care OR homecare OR house call).mp. [mp=title, abstract, heading word, drug trade name, original title, device manufacturer, drug manufacturer, device trade name, keyword heading word, floating subheading word, candidate team word]  4=(health status OR health outcome* OR disabilit* OR impair* OR frail* OR health related quality of life OR HRQoL OR quality of life OR unmet need* OR met need* OR need*).mp. [mp=title, abstract, heading word, drug trade name, original title, device manufacturer, drug manufacturer, device trade name, keyword heading word, floating subheading word, candidate team word]  5= 1 and 2 and 3 and 4 | 567 |
| Emcare | 1=(older adult* OR older people OR older person* OR aged OR elder* OR elder* people OR elder* person* OR senior citizen OR old* citizen).mp. [mp=title, abstract, heading word, drug trade name, original title, device manufacturer, drug manufacturer, device trade name, keyword heading word]  2=(resilien* OR coping OR adversit* OR bounce back OR positive adaptation OR protective factor*).mp. [mp=title, abstract, heading word, drug trade name, original title, device manufacturer, drug manufacturer, device trade name, keyword heading word]  3=(preventive home visit* OR home visit* OR home care OR homecare OR house call).mp. [mp=title, abstract, heading word, drug trade name, original title, device manufacturer, drug manufacturer, device trade name, keyword heading word]  4=(health status OR health outcome* OR disabilit* OR impair* OR frail* OR health related quality of life OR HRQoL OR quality of life OR unmet need* OR met need* OR need*).mp. [mp=title, abstract, heading word, drug trade name, original title, device manufacturer, drug manufacturer, device trade name, keyword heading word]  5= 1 and 2 and 3 and 4 | 198 |
| PsycINFO | 1=(older adult* OR older people OR older person* OR aged OR elder* OR elder* people OR elder* person* OR senior citizen OR old* citizen).mp. [mp=title, abstract, heading word, table of contents, key concepts, original title, tests & measures, mesh word]  2=(resilien* OR coping OR adversit* OR bounce back OR positive adaptation OR protective factor*).mp. [mp=title, abstract, heading word, table of contents, key concepts, original title, tests & measures, mesh word]  3=(preventive home visit* OR home visit* OR home care OR homecare OR house call).mp. [mp=title, abstract, heading word, table of contents, key concepts, original title, tests & measures, mesh word]  4=(health status OR health outcome* OR disabilit* OR impair* OR frail* OR health related quality of life OR HRQoL OR quality of life OR unmet need* OR met need* OR need*).mp. [mp=title, abstract, heading word, table of contents, key concepts, original title, tests & measures, mesh word] | 241 |
| Cochrane Library | "older adult*" OR "older people" OR "older person*" OR aged OR elder* OR "elder* people" OR elder* person* OR "senior citizen" OR "old* citizen" in Title Abstract Keyword AND resilien* OR coping OR adversit* OR "bounce back" OR "positive adaptation" OR "protective factor*" in Title Abstract Keyword AND "preventive home visit*" OR "home visit*" OR "home care" OR homecare OR "house call" in Title Abstract Keyword AND "health status" OR "health outcome*" OR disabilit* OR impair* OR frail* OR "health related quality of life" OR HRQoL OR "quality of life" OR "unmet need*" OR "met need*" OR need* in Title Abstract Keyword | 150 |
| Google Scholar | (("older adult*" OR "older person*" OR aged OR elder*) AND ("preventive home visit* ") AND (resilien* OR health OR “quality of life”)) | 61 |
| Google | (("older adult*" OR "older person*" OR aged OR elder*) AND ("preventive home visit* ") AND (resilien* OR health OR “quality of life”)) | 13 |
